# OXY-SCORE and Volatile Anesthetics: A New Perspective of Oxidative Stress in EndoVascular Aneurysm Repair—A Randomized Clinical Trial

**DOI:** 10.3390/ijms251910770

**Published:** 2024-10-07

**Authors:** Alba Burgos-Santamaría, Pilar Rodríguez-Rodríguez, Ana Arnalich-Montiel, Silvia M. Arribas, Carmen Fernández-Riveira, I. María Barrio-Pérez, Javier Río, José Manuel Ligero, Begoña Quintana-Villamandos

**Affiliations:** 1 Department of Anesthesia and Intensive Care, Gregorio Marañón’s University Hospital, 28007 Madrid, Spain; anaarn01@ucm.es (A.A.-M.); cfernandezr@salud.madrid.org (C.F.-R.); irmamaria.barrio@scsalud.es (I.M.B.-P.); maquin01@ucm.es (B.Q.-V.); 2 Department of Physiology, Faculty of Medicine, Autónoma University, 28029 Madrid, Spain; pilar.rodriguezr@uam.es (P.R.-R.); silvia.arribas@uam.es (S.M.A.); 3 Department of Angiology and Vascular Surgery, Gregorio Marañón’s University Hospital, 28007 Madrid, Spain; javier.rio@salud.madrid.org (J.R.); josemanuel.ligero@salud.madrid.org (J.M.L.); 4 Department of Pharmacology and Toxicology, Faculty of Medicine, Complutense University, 28040 Madrid, Spain

**Keywords:** oxidative stress, volatile anesthetics, aortic aneurysm, OXY-SCORE

## Abstract

An aortic aneurysm (AA) is a life-threatening condition. Oxidative stress may be a common pathway linking multiple mechanisms of an AA, including vascular inflammation and metalloproteinase activity. Endovascular aneurysm repair (EVAR) is the preferred surgical approach for AA treatment. During surgery, inflammation and ischemia–reperfusion injury occur, and reactive oxygen species (ROS) play a key role in their modulation. Increased perioperative oxidative stress is associated with higher postoperative complications. The use of volatile anesthetics during surgery has been shown to reduce oxidative stress. Individual biomarkers only partially reflect the oxidative status of the patients. A global indicator of oxidative stress (OXY-SCORE) has been validated in various pathologies. This study aimed to compare the effects of the main volatile anesthetics, sevoflurane and desflurane, on oxidative status during EVAR. Eighty consecutive patients undergoing EVAR were randomized into two groups: sevoflurane and desflurane. Plasma biomarkers of oxidative damage (protein carbonylation and malondialdehyde) and antioxidant defense (total thiols, glutathione, nitrates, superoxide dismutase, and catalase activity) were measured before surgery and 24 h after EVAR. The analysis of individual biomarkers showed no significant differences between the groups. However, the OXY-SCORE was positive in the desflurane group (indicating a shift towards antioxidants) and negative in the sevoflurane group (favoring oxidants) (*p* < 0.044). Compared to sevoflurane, desflurane had a positive effect on oxidative stress during EVAR. The OXY-SCORE could provide a more comprehensive perspective on oxidative stress in this patient population.

## 1. Introduction

An aortic aneurysm (AA) is defined as a permanent dilation of the aorta and is a common, life-threatening condition. Important risk factors for an AA include male sex, advanced age, family history of AA, smoking, atherosclerosis, hypercholesterolemia, and hypertension [1,2,3]. Most patients are asymptomatic at the time of diagnosis. If rupture occurs, the risk of death can be as high as 81%. While no specific medical therapy has been found to halt aneurysm expansion, strict control of cardiovascular risk factors is recommended [4,5].

Elective surgical treatment of an AA depends on the symptoms, the aneurysm size, and the patient’s operative risk [6]. Open aortic surgery is an invasive procedure that involves making an incision in the patient’s abdomen to access the aorta. Endovascular surgery has offered a major advance in the treatment of AAs. Compared to open surgical repair, endovascular aneurysm repair (EVAR) is a less invasive treatment. In EVAR, stent grafts are delivered percutaneously through the femoral artery. The stent graft is employed to seal the sac from the inside of the aneurysm, leaving the aneurysm wall intact. EVAR improves perioperative morbidity, mortality, and recovery compared to traditional open aortic surgery [7,8].

EVAR can be performed under local, locoregional, or general anesthesia. Data on the preferred type of anesthesia for elective EVAR are limited, and clinical practice guidelines support that practice should follow local hospital routine and individual patient assessment [5,9]. When general anesthesia is used, sevoflurane and desflurane are the most commonly used anesthetic agents for its maintenance.

Oxidative stress is defined as an imbalance between pro-oxidants and antioxidants, in favor of the former, that leads to potential molecular damage [10]. A balance between reactive oxygen species (ROS) and antioxidants is necessary for the normal cellular function. Increased ROS levels can damage macromolecules such as DNA, lipids, and proteins and have been implicated in the etiology of various diseases, especially cardiovascular conditions [11,12]. Oxidative stress plays a key role in the pathogenesis of an AA. ROS production is induced by mechanical stress on the vascular wall. The overproduction of ROS may induce inflammation, matrix metalloproteinase activity, and vascular smooth muscle cell apoptosis. Additionally, oxidative stress is associated with clinical risk factors in AA (smoking, atherosclerosis, and hypertension) and aneurysm size [13,14,15,16].

Therefore, preoperative patients undergoing EVAR present elevated levels of oxidative stress. Moreover, intraoperative oxidative stress contributes to the acute phase response during tissue injury. During surgery, inflammation and ischemia–reperfusion injury occur, with ROS playing a key role in their modulation [17]. The extent of the surgical procedure is a determinant of intraoperative oxidative stress, with more invasive procedures associated with higher oxidative stress levels [18,19,20]. In addition, the anesthetic agents used to maintain anesthesia during surgery can influence oxidative stress. Anesthesiologists have a variety of pharmaceutical agents in the operating room, with sevoflurane and desflurane being the most commonly used volatile anesthetics for the maintenance of general anesthesia. Furthermore, postoperative increases in oxidative stress are linked to worse clinical outcomes, including postoperative complications such as cardiovascular death, atrial fibrillation, heart failure, and prolonged recovery [21]. Thus, perioperative stress is a complex response involving patient, surgical, and anesthetic factors [17].

Given the accumulating evidence of the impact of anesthetic agents on oxidative stress, during the last years, numerous studies have examined the role of volatile anesthetics on oxidative stress during surgery. Sevoflurane and desflurane may provide anesthetic-induced preconditioning (APC). APC refers to the phenomenon where brief exposure to volatile anesthetics induces a protective state in tissues, making them more resistant to subsequent ischemic or reperfusion injury. This preconditioning effect is achieved through various molecular pathways (e.g., mitochondrial KATP channels, δ-opioid and bradykinin receptors, nitric oxide synthase, and superoxide dismutase) [22].

APC comprises two distinct phases: an early preconditioning (1–6 h after anesthetic exposure) and then a late phase (24–72 h) [22]. During the late phase of APC, antioxidants mechanisms predominate with an increase in the expression and activity of antioxidant enzymes such as superoxide dismutase (SOD), catalase, and glutathione peroxidase. This late phase of APC appears to contribute to the long-term protective effects of preconditioning and support cellular resilience against oxidative stress [23,24,25]. These cardiac benefits have been widely studied in cardiac surgery. For this reason, volatile anesthetics have been the hypnotics of choice in cardiac surgery for decades, resulting in reductions in myocardia injury, length of hospital stay, and mortality [26,27]. Sevoflurane and desflurane-induced preconditioning have been shown to be mediated by ROS in isolated the human right atria in vitro [28]. The cardioprotective effects of sevoflurane and desflurane have been studied in aortic surgery, although the evidence in this area is scarce and inconclusive [29].

There are not many studies assessing the pro-oxidant/antioxidant effects of sevoflurane and desflurane. Moreover, these studies are conducted in different surgical procedures and measure varying endpoints using blood samples [30,31,32,33,34,35]. Additionally, most studies focus on individual biomarkers without considering that oxidative stress is a dynamic and complex process. The measurement of particular biomarkers may not accurately reflect the overall oxidative status of the patient. Several proposals have been published to evaluate pro-oxidant/antioxidant homeostasis with the goal of obtaining a global index and avoiding the bias of individual biomarkers [36].

The OXY-SCORE was proposed by Veglia et al. [37] in 2006, showing how the combination of different biomarkers can provide a powerful index for evaluating oxidative stress in relation to gender, age, and coronary artery disease. To calculate the OXY-SCORE, the authors described a damage score (DS) and a protection score (PS). The DS was the result of individual measurements in plasma of free and total malondialdehyde (MDA), oxidized/reduced glutathione (GSSG/GSH), and urinary isoprostane-PF2α-III levels. The PS included GSH, α- and γ-tocopherol, and individual antioxidant capacity. Since its initial proposal, different modified versions of the OXY-SCORE have been used in preclinical models [38,39] and human studies [37,40,41,42,43]. The OXY-SCORE has become a valuable tool in evaluating cardiovascular diseases.

Therefore, oxidative stress is significantly increased preoperatively in patients with AAs. In addition, oxidative stress plays a crucial role in surgical stress and is implicated in postoperative recovery and mortality. Consequently, its reduction through anesthesia management (using agents with potential antioxidant properties such as volatile anesthetics) may contribute to improved patient outcomes.

To the best of our knowledge, no previous studies have assessed the effect of volatile anesthetics on the pro-oxidant/antioxidant balance using a global index during EVAR. The aim of this study is to examine the impact of volatile anesthetics, sevoflurane, and desflurane on the oxidative status of patients undergoing elective EVAR. We propose an OXY-SCORE that incorporates individual plasma biomarkers of oxidative damage and antioxidant defense, allowing us to compare the effects of sevoflurane and desflurane on the pro-oxidant/antioxidant balance during EVAR.

## 2. Results

### 2.1. Demographic Data

**Figure 1:**
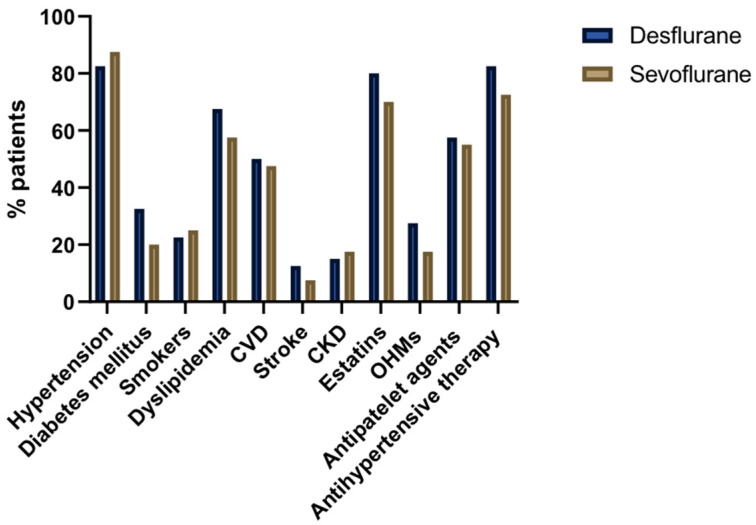
Comorbidities and chronic medical treatments in sevoflurane and desflurane groups. Data are expressed as frequency (percentages). CKD: chronic kidney disease; CVD: cardiovascular disease; OHMs: oral hypoglycemic medications.

A total of 80 patients were consecutively recruited and randomized into two groups based on the volatile anesthetic administered during surgery: sevoflurane (n = 40) and desflurane (n = 40). In the sevoflurane group, 39 patients were male, with a mean age of 76.35 ± 5.62 years and a mean weight of 81.34 ± 12.42 kg. In the desflurane group, all 40 patients were male, with a mean age of 76.75 ± 6.99 years and a mean weight of 85.05 ± 15.44 kg. There were no significant differences in age (*p* = 0.674), gender (*p* = 1.000), or weight (*p* = 0.240) between the sevoflurane and desflurane groups. The comorbidities and chronic medical treatments of the patients are displayed in Figure 1. Statistical analysis of these variables did not show differences between the sevoflurane and desflurane groups: Hypertension (87.5% vs. 82.5%), diabetes mellitus (20% vs. 32.5%), nonsmoker, former smoker > 6 months (77.5% vs. 75%), former smoker < 6 months (5% vs. 5%), smoker (17.5% vs. 20%), cholesterol levels (153.96 ± 33.37 vs. 156.87 ± 43.06 mg/dL), cardiovascular disease (47.5% vs. 50%), stroke (7.5% vs. 12.5%) and chronic kidney disease (17.5% vs. 15%).

### 2.2. Anesthetic and Surgical Variables

Anesthetic and surgical variables are summarized in Table 1. No significant differences were observed between the desflurane and sevoflurane groups in terms of the anesthetic and surgical variables. Eighty patients (40 in desflurane group and 40 in the sevoflurane group) with AAs underwent EVAR.

### 2.3. Individual Biomarkers of Oxidative Stress

Biomarkers of oxidative damage, including protein carbonylation, malondialdehyde (MDA), and antioxidant defense—including total thiols, reduced glutathione (GSH), nitrates, SOD, and catalase activity—were measured in plasma samples. Plasma individual biomarkers were assessed in both groups before surgery (moment 0) and 24 h after EVAR (moment 1). There were no statistically significant differences in individual biomarkers between sevoflurane and desflurane groups at either moment 0 or moment 1 (Table 2 and Table 3).

The plasma individual biomarkers of oxidative damage and antioxidant defense were assessed at 24 h postsurgery and compared to baseline levels for each study group separately.

In the sevoflurane group, protein carbonylation did not show significant differences (*p* = 0.809) between the baseline and 24 h postsurgery. However, the MDA levels decreased significantly 24 h after surgery (*p* = 0.018). In terms of antioxidant defense, the plasma concentration of nitrates (*p* = 0.0001) and catalase activity (*p* = 0.0001) increased 24 h post-EVAR. There were no differences in the total thiol concentration (*p* = 0.914). Conversely, there was a decrease in the GSH concentration (*p* = 0.0001) and SOD activity (*p* = 0.0001) 24 h after sevoflurane administration.

In the desflurane group, protein carbonylation did not show significant differences (*p* = 0.106) between moment 0 and moment 1. The MDA levels decreased significantly 24 h post-EVAR (*p* = 0.027). Increases in plasma nitrates (*p* = 0.0001) and catalase activity (*p* = 0.0001) were observed 24 h after surgery, while the total thiol levels remained stable (*p* = 0.745). There was a significant decrease in the GSH levels (*p* = 0.0001) and SOD activity (*p* = 0.0001) 24 h after surgery and desflurane administration.

### 2.4. OXY-SCORE

To calculate a global index of oxidative stress in both groups (sevoflurane and desflurane), individual biomarkers of oxidative damage and antioxidant defense were measured before surgery (moment 0) and 24 h after EVAR (moment 1). The OXY-SCORE was calculated using the methodology previously described by Veglia et al. [37]. The damage score (DS) was computed as the average of the standardized biomarkers of oxidative damage (protein carbonyation and MDA). The protection score (PS) was calculated by averaging the standardized biomarkers of antioxidant defense (total thiols, nitrates, GSH, SOD, and catalase activity). The OXY-SCORE was obtained by subtracting the PS from the DS. Positive values suggest an imbalance favoring antioxidants, while negative values indicate predominant oxidative damage.

At moment 0, the OXY-SCORE was near zero in both the sevoflurane (−0.0048977 ± 0.378) and desflurane groups (−0.0001729 ± 0.318). No significant differences were observed in the OXY-SCORE between the two groups before surgery (*p* = 0.992). Although close to zero, the negative values in both groups at the baseline indicate an imbalance toward oxidative damage in the 80 patients at the preoperative period.

**Figure 2:**
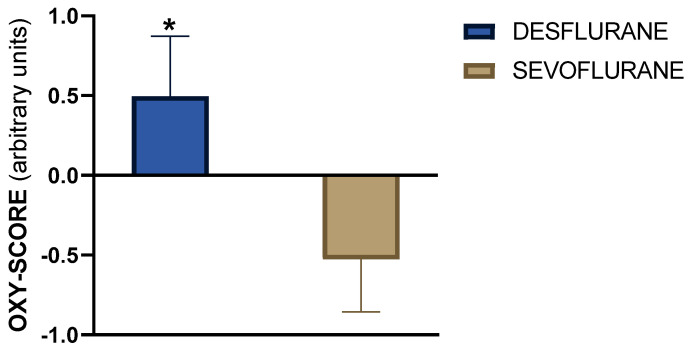
OXY-SCORE 24 h after surgery (moment 1) in sevoflurane and desflurane groups. A statistically significant difference between sevoflurane and desflurane group is shown (* *p* < 0.05). Data are expressed as mean ± SEM.

At moment 1, 24 h after exposure to the volatile anesthetic, the OXY-SCORE varied between the two groups (Figure 2). The OXY-SCORE was negative in patients exposed to sevoflurane (−0.526281 ± 0.330) and positive in patients anesthetized with desflurane (0.495972 ± 0.376). The negative value in patients who received sevoflurane suggests a predominance of oxidative damage in this group, while the positive value in patients who received desflurane indicates an imbalance toward antioxidants. There was a statistically significant difference in the OXY-SCORE calculated 24 h after EVAR between the sevoflurane and desflurane groups (*p* < 0.044).

## 3. Discussion

To our knowledge, this clinical trial is the first to demonstrate the superiority of desflurane over sevoflurane regarding the oxidative status of patients undergoing elective EVAR, as assessed by a global index (OXY-SCORE).

Oxidative stress plays an important role in the pathogenesis of AAs [13,14,15,16] and during surgical treatments such as open repair and EVAR [19]. In recent years, the impact of volatile anesthetics on oxidative stress during surgery has been investigated [30,31,32,33,34,35,44]. However, no prior studies have compared the effects of sevoflurane and desflurane on the redox status of patients undergoing endovascular aortic surgery.

Several factors can influence the oxidative status of patients, including the patient’s preoperative condition, surgical technique [20], general anesthesia [45], and episodes of intraoperative hypotension [46]. In our study, demographic variables, comorbidities, surgical technique (EVAR), and hemodynamic management did not significantly differ between the sevoflurane and desflurane groups. Therefore, the observed differences in oxidative status are likely attributable to the different volatile anesthetics used during the surgical period (sevoflurane or desflurane).

Volatile anesthetics have been proposed to possess cardioprotective properties through the induction of APC in in preclinical models [47,48] and human studies [28,49,50,51]. Both sevoflurane and desflurane provide cardioprotection via several mechanisms, with one of them being decreasing ROS production and reducing oxidative stress [26,49,51,52]. In a rat model, desflurane demonstrated a more potent effect in stimulating APC mechanisms compared to sevoflurane [48]. Although volatile anesthetics are commonly used during vascular surgery, their specific cardioprotective effects in aortic surgery remain uncertain [29,53,54,55].

The role of sevoflurane and desflurane in oxidative stress remains controversial. Various preclinical and clinical models have assessed their impact on redox status during surgery, employing diverse sample collection methods, experimental protocols, and biomarkers [56,57]. For instance, in a mechanically ventilated swine model, desflurane increased serum and pulmonary lavage MDA, whereas sevoflurane had a minimal effect [58]. An increase in plasma lipid peroxidation following desflurane administration has also been reported in healthy patients undergoing laparoscopic surgery [30]. In contrast, our study showed a significant decrease in the MDA levels 24 h post-EVAR in the desflurane group. Similarly, Türkan et al. [59] demonstrated a reduction in the MDA concentration in liver tissue after desflurane administration. They also evaluated oxidative stress in various tissues following exposure to both volatile anesthetics, noting decreased SOD and GSH levels in liver and kidney tissues, respectively. Other authors have reported sevoflurane-induced intracellular GSH depletion in neutrophils [60] and decreased SOD activity in erythrocytes [31], which are consistent with our findings of reduced GSH and SOD levels 24 h after surgery in both groups.

Therefore, the literature on volatile anesthetics’ impact on oxidative stress remains controversial. The effect of sevoflurane and desflurane on the pro-oxidant/antioxidant balance may depend on factors such as patient comorbidities, surgical procedures, and specific organs studied. Furthermore, most studies have focused on individual biomarkers of oxidative stress.

In our study, individual biomarker assessments did not reveal significant differences between the sevoflurane and desflurane groups. This is consistent with the previous literature suggesting that individual biomarkers provide only a partial insight into a patient’s oxidative status [56]. Given the complex pathophysiology of cardiovascular diseases, no single biomarker can distinguish between health and disease. Therefore, oxidative stress indexes have been proposed to combine multiple biomarkers of oxidative damage and antioxidant defense into a single value for a more integrated evaluation of a patient’s oxidative status [36].

It is noteworthy that not all published oxidative stress indexes are reliable or useful in clinical practice. In 2019, Sanchez-Rodriguez et al. [36] reviewed oxidative stress indexes for diagnosing health or disease in humans. They concluded that the OXY-SCORE is a reliable, practical, and effective predictor of clinically relevant outcomes. The OXY-SCORE can be adapted to different pathologies by adding or removing variables from its formula [37], making it a versatile tool for evaluating the impact of volatile anesthetics on the oxidative status of patients undergoing EVAR.

Previous studies have demonstrated that the OXY-SCORE might be useful for assessing oxidative stress in various cardiovascular diseases. The OXY-SCORE has been utilized to detect oxidative alterations in models of fetal undernutrition and hypertension development [61], as well as to monitor the cardiovascular protective effects of dronedarone [39]. In clinical models, Veglia et al. [37] demonstrated the OXY-SCORE’s high sensitivity and specificity in identifying healthy individuals across different demographics and its effectiveness in diagnosing patients with coronary artery disease (area under the receiver operating characteristic [ROC] curve 0.96). They also [40] compared the OXY-SCORE with individual biomarkers in assessing oxidative stress in patients undergoing coronary artery bypass graft surgery, where the OXY-SCORE offered a more comprehensive assessment of redox balance and accurately distinguished patients with or without cardiopulmonary bypass (area under the ROC curve 0.90). The index has been used in the early diagnosis of venous insufficiency (OXyVen) [41] and albuminuria in early stages of chronic kidney disease (AlbuminOXY-SCORE) [42]. Recently, our study group designed an OXY-SCORE that could aid in the diagnosis of left ventricular hypertrophy and monitor the treatment response (area under the ROC curve 0.742) [43].

The choice of biomarkers incorporated in the global index should be guided by the study goal and clinical relevance to the patient [56]. The OXY-SCORE has not only been used for diagnosing health or disease but also for evaluating treatment effectiveness, such as dronedarone in coronary artery remodeling [39]. With the same objective in mind, we performed an OXY-SCORE using plasma biomarkers that have previously been demonstrated to be valid surrogates of cardiovascular tissue [39,61,62,63,64,65] to evaluate the effect of volatile anesthetics on oxidative stress following endovascular aortic surgery.

At moment 0, the OXY-SCORE was negative in both the sevoflurane and desflurane groups, indicating a preoperative imbalance favoring oxidative damage in all 80 patients. This pro-oxidant status before EVAR surgery aligns with previous studies showing high preoperative levels of MDA and protein carbonylation [20,66]. This oxidative damage at the baseline may be explained by the oxidative stress associated with the patients, as an endovascular approach is typically indicated for high-risk individuals [5].

However, at moment 1–24 h after exposure to sevoflurane and desflurane—significant differences in the OXY-SCORE emerged between groups (*p* < 0.044). The desflurane group had a positive OXY-SCORE (imbalance towards antioxidants), while the sevoflurane group showed a negative OXY-SCORE (imbalance towards oxidants). Although the differences in OXY-SCORE values 24 h after surgery were small, they were statistically significant and may have potential implications for patient outcomes. In earlier research, subtle variations in OXY-SCORE values were observed and considered relevant, wherein even minor variations in OXY-SCORE values can have clinical relevance [37,40,41,43].

Our findings suggest that while individual biomarkers did not differ significantly between the sevoflurane and desflurane groups, the OXY-SCORE calculated 24 h post-EVAR showed significant group differences. Desflurane seemed to improve the plasma redox status in patients undergoing elective EVAR. These findings suggest that the OXY-SCORE may offer a more comprehensive assessment of the oxidative status of patients undergoing EVAR.

The study has some limitations. First, it was not possible to blind the study to the anesthesiologist who administered the volatile anesthetic during the surgery (different vaporizers). The other professionals involved in the study remained blind to the volatile anesthetic administered. Second, another potential limitation is that there was a lack of consensus concerning which biomarker should be measured in each disease [57]. Numerous biomarkers of oxidative stress have been employed to study different disease and are often measured using distinct methods. No single biomarker can distinguish between health and disease and reflects only partially the redox status of the patients. In the present study, we decided to measure plasmatic biomarkers that have previously demonstrated to be a valid surrogate of cardiovascular tissue and that our study group had experience in their determination [39,61,62,63,64,65]. Finally, clinical outcomes cannot be analyzed in this study. The aim of the present study was to compare the effect of sevoflurane and desflurane over the oxidative status of patients undergoing elective EVAR. The sample size was calculated for detecting significant changes in the study of oxidative stress. It would be interesting to assess if this alterations in oxidative stress with sevoflurane and desflurane have a correlation with clinical outcomes in order to change our clinical practice in the operating room.

## 4. Materials and Methods

### 4.1. Study Design

Eighty patients were consecutively recruited between March 2017 and September 2021 after being recommended for elective AA and scheduled for EVAR. The indications for aneurysm repair, based on guidelines, included symptomatic aneurysm, aneurysm size ≥ 5.5 cm in men, ≥5.0 cm in women, or when rapid aneurysm growth (>1 cm/year) [5]. Preoperative computed tomography angiography was performed for treatment planning.

**Figure 3:**
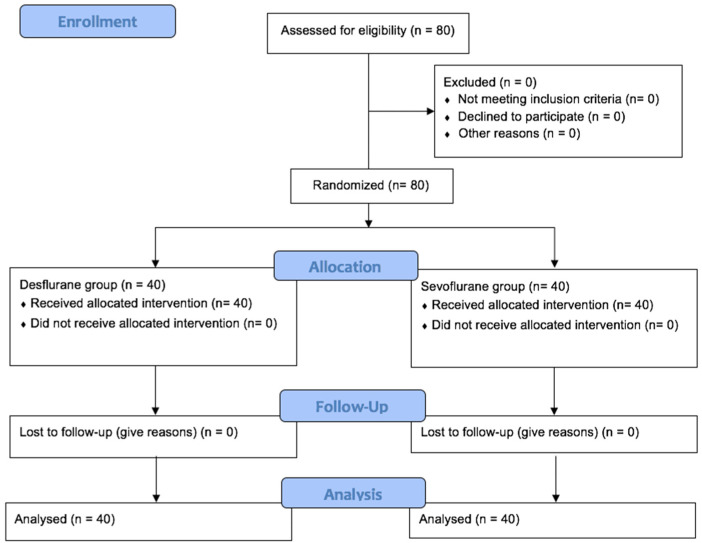
Flow diagram (Consolidated Standards of Reporting Trials [CONSORT] chart).

Inclusion criteria included adult patients with diagnosis of AA undergoing elective EVAR who had signed informed consent. Patients were excluded if the use of sevoflurane or desflurane was contraindicated or if informed consent was not obtained. Patients were randomized into two groups (1:1 ratio) based on the volatile anesthetic used during surgery: sevoflurane group (n = 40) and desflurane group (n = 40) (Figure 3). Random allocation sequence was performed with EPIDAT program.

During the intraoperative period, monitoring included pulse oximetry, electrocardiogram, arterial catheterization (radial artery), Bispectral Index (BIS^TM^) to assess the depth of anesthesia, and Train of Four (ToF) to assess the degree of neuromuscular blockade. Fentanyl (2 μg/kg), propofol (2–3 mg/kg), and rocuronium (0.6–1.2 mg/kg) were used for induction of general anesthesia. Maintenance of general anesthesia was achieved with sevoflurane or desflurane based on the randomization. The concentration of volatile anesthetics administered during surgery followed the data sheets of sevoflurane and desflurane.

BIS monitor incorporates frequency domain, time domain, and bispectral analysis of the electroencephalogram and is displayed as a dimensionless number ranging from 0 (deep anesthesia) to 100 (awake), with a target range of 40–60 for surgical anesthesia. BIS has been correlated well with hypnotic state and anesthetic drug concentration [67]. Sevoflurane and desflurane were titrated to maintain the BIS target range of 40–60. Additional doses of rocuronium (0.25 mg/kg) were used during surgery to maintain patient immobility. Once the surgery was completed, 1 g of paracetamol was administered for analgesia. Neuromuscular blockade was assessed using ToF and, if necessary, reversed with sugammadex. Patients’ lungs were ventilated with an FiO2 ≥ 0.4 using tidal volumes of 6–8 mL/kg (ideal body weight) and respiratory rate to maintain an end-tidal CO_2_ between 35–45 mmHg. Maintenance fluid therapy was administered using crystalloids at 2–4 mL/kg/h. Hemodynamic management followed the protocol described by Pestaña et al. [68], which included fluid administration and vasoactive drugs based on arterial blood pressure, cardiac index, and stroke volume response. After extubation in the operating room, patients were transferred to postoperative care unit. Patients were discharged from the postoperative care unit 24 h after surgery, and follow-up was completed.

Arterial blood samples (collected in EDTA tube) were drawn in the operating room before anesthesia induction (referred to as “moment 0”) and 24 h after surgery (referred to as “moment 1”). The blood sample at moment 0 reflects the patient’s preoperative oxidative stress. The blood sample at moment 1 was collected 24 h after surgery and cessation of volatile anesthetic administration. We collected blood samples 24 h after exposure to sevoflurane or desflurane to study the effect of late-phase APC. Additionally, most studies measuring oxidative stress in aortic surgery obtain blood samples before and after anesthesia at various time intervals up to 24–48 h [19].

Blood samples were separated into plasma and erythrocytes by centrifugation at 2000× *g* for 15 min at 4 °C. Plasma samples were stored at −80 °C until assays were performed.

This study is part of a clinical trial registered with ClinicalTrials.gov under the identifier NCT: 03917186. The study was approved by the Comité de Ética de la Investigación con Medicamentos, Gregorio Marañón’s University Hospital, Madrid, Spain (Project identification code. EudraCT: 2016-003906-16, 8 February 2017).

### 4.2. Data Collection

Patients’ demographic characteristics, including age, gender, and weight, were recorded. Comorbidities such as hypertension, diabetes mellitus, smoking, dyslipidemia, cardiovascular disease, stroke, chronic kidney disease, and chronic medical treatment were also noted. Anesthetic variables (anesthesia duration, volatile anesthetic used, duration of sevoflurane/desflurane administration, fluid input, and use of vasoactive drugs) and surgical variables (operative time and aneurysm size), were recorded, along with preoperative and postoperative hemoglobin levels.

### 4.3. Study of Oxidative Stress

Biomarkers of oxidative damage (protein carbonylation and MDA) and antioxidant defense (total thiols, GSH, nitrates, SOD, and catalase activity) were measured in plasma samples. Absorbance was read on a microplate reader (Synergy HT Multimode; BioTek, Layton, UT, USA) using bovine serum albumin as the standard.

#### 4.3.1. Biomarkers of Oxidative Stress

Protein carbonylation (nmol/mg protein) assay was performed using 2,4-dinitrophenylhydrazine (DNPH, Sigma Aldrich, Madrid, Spain), as previously described by other authors [69]. Protein content was assessed by Coomassie-blue-based microtiter plate assay according to manufacturer’s instructions (Bio-Rad, Madrid, Spain). Absorbance was measured at 370 nm.

Malondialdehyde (MDA) (μM) levels were measured using the Lipid Peroxidation-LPO Assay Kit (BioQuoChem, Gijon, Spain) following the manufacturer’s instructions. Absorbance was measured at 586 nm.

Total thiols (μmol/mg protein) were quantified using 5,5′-dithiobis(2-nitrobenzoic acid) (DTNB assay with Ellman’s reagent) [69]. The plate was read at 412 nm, and thiol content was expressed as nanomolar of reduced glutathione per milligram of protein.

#### 4.3.2. Biomarker of Antioxidant Defense

Reduced glutathione (GSH) (μmol/mg protein) was assessed using a fluorometric method based on the reaction with o-phthalaldehyde (OPT) [70]. This fluorescence can be measured (λ_excitation_ 360 ± 40 and λ_emission_ wavelengths 460 ± 40 nm).

Nitrates (μM) measurement was performed using the protocol previously described by Miranda et al. [71]. The plate was read at 540 nm.

Superoxide dismutase activity (SOD) (mU SOD/mg protein) was assessed using the SOD Activity Assay (BioQuoChem) according to the manufacturer’s instructions. Absorbance was measured at 450 nm.

Catalase activity (U catalase/mg protein) was assessed using the Catalase Activity Assay (BioQuoChem, Gijon, Spain) following the manufacturer’s instructions. Absorbance was measured at 540 nm.

### 4.4. Calculation of OXY-SCORE

To calculate the OXY-SCORE, individual biomarkers of oxidative damage and antioxidant defense before surgery (moment 0) and 24 h after EVAR (moment 1) were used. Veglia et al. [37] initially described the feasibility of adding or removing variables from their formula. The plasma biomarkers measured in this study for calculating OXY-SCORE showed good correlation with their levels in tissue in prior research. Additionally, various authors have used these biomarkers to calculate the OXY-SCORE in different CVDs such as hypertension [38], venous insufficiency [41], and LVH [43].

The OXY-SCORE was calculated using the methodology previously described by Veglia et al. [37]. *Kolmogorov–Smirnov* tests were used to assess the normal distribution of biomarkers; those not normally distributed were log-transformed. Subsequently, individual biomarkers were standardized to account for different measurement units. Damage score (DS) and protection score (PS) were calculated using the individual biomarkers of oxidative damage and antioxidant defense, respectively. The OXY-SCORE was calculated for both the sevoflurane and desflurane groups using the following equation: [OXY-SCORE = Mean (PSi − DSi)*n*], where *n* is the study group and *i* is the individual. The resulting OXY-SCORE is expressed in arbitrary units (AU).

Positive and negative OXY-SCORE values may be obtained, with zero indicating an ideal balance between pro-oxidants and antioxidants. Positive values indicate a predominance of antioxidants, whereas negative values suggest prevailing oxidative damage.

### 4.5. Statistical Analysis

In quantitative variables, data were described as mean, standard error of mean (SEM), and standard deviation (SD). In qualitative variables, data were expressed in terms of relative frequency (%). Categorical variables were tested using Fischer’s exact test. An independent Student’s T test was applied to test the difference between treatment in each moment. The intraindividual variable was calculated by subtraction moment 0 to moment 1 and was tested using Student’s T test as well. In all analysis, a *p*-value (*p*) < 0.05 was considered as statistically significant.

## 5. Conclusions

Our findings suggest that desflurane may have a more favorable impact on oxidative stress during EVAR compared to sevoflurane. The OXY-SCORE appears to provide a valuable approach for assessing oxidative stress in vascular surgery and offers a more holistic view of oxidative stress than individual biomarkers. While our study highlights the potential utility of the OXY-SCORE in assessing oxidative stress in patients undergoing elective EVAR, further research is necessary to elucidate its clinical utility.

## Figures and Tables

**Table 1:** Anesthetic and surgical variables.

	Desflurane Group (n = 40)	Sevoflurane Group (n = 40)	*p* Value
Aneurysm size (mm)	60.27 ± 10.69	59.90 ± 5.61	0.848
Operative time (min)	182.38 ± 79.16	156.55 ± 59.31	0.103
Anesthesia duration (min)	250.28 ± 87.15	220.28 ± 64.83	0.091
Duration of sevoflurane/desflurane administration (min)	230.93 ± 86.80	197.85 ± 61.91	0.053
Hemoglobin (g/dL)			
Moment 0	13.73 ± 1.56	13.38 ± 1.48	0.320
Moment 1	11.71 ± 1.44	11.98 ± 1.52	0.423
Blood transfusion (%)	1 (2.5%)	3 (7.5%)	0.610
Lactate (mmol/L)			
Moment 0	1.29 ± 0.40	1.40 ± 0.51	0.305
Moment 1	1.38 ± 0.86	1.28 ± 0.34	0.495

Data are expressed as mean ± SD, except blood transfusion variable, which is expressed as the percentage of patients.

**Table 2:** Biomarkers of oxidative damage and antioxidant defense before surgery (moment 0).

Plasma Biomarker	Desflurane Group (n = 40)	Sevoflurane Group (n = 40)	*p* Value
Protein carbonylation (nmol/mg protein)	3.869 ± 0.349	3.459 ± 0.395	0.441
MDA (μM)	18.681 ± 2.013	17.487 ± 0.824	0.592
Nitrates (μM)	39.230 ± 4.296	49.153 ± 4.306	0.107
Total thiols (μmol/mg protein)	0.008 ± 0.001	0.007 ± 0.001	0.568
GSH (μmol/mg protein)	0.490 ± 0.088	0.319 ± 0.047	0.092
SOD (mU SOD/mg protein)	33.992 ± 1.625	37.105 ± 2.615	0.328
Catalase (U catalase/mg protein)	7.042 ± 0.496	7.116 ± 0.809	0.944

Data are expressed as mean ± SEM. MDA: malondialdehyde; GSH: reduced glutathione; SOD: superoxide dismutase activity.

**Table 3:** Biomarkers of oxidative damage and antioxidant defense after surgery (moment 1).

Plasma Biomarker	Desflurane Group (n = 40)	Sevoflurane Group (n = 40)	*p* Value
Protein carbonylation (nmol/mg protein)	3.206 ± 0.217	3.507 ± 0.227	0.343
MDA (μM)	14.012 ± 0.655	14.495 ± 0.861	0.657
Nitrates (μM)	94.925 ± 5.688	88.695 ± 5.090	0.419
Total thiols (μmol/mg protein)	0.008 ± 0.001	0.008 ± 0.001	0.249
GSH (μmol/mg protein)	0.083 ± 0.004	0.083 ± 0.003	0.996
SOD (mU SOD/mg protein)	24.381 ± 0.697	23.549 ± 0.592	0.366
Catalase (U catalase/mg protein)	10.851 ± 0.278	10.793 ± 0.291	0.885

Data are expressed as mean ± SEM. MDA: malondialdehyde; GSH: reduced glutathione; SOD: superoxide dismutase activity.

## Data Availability

Data is contained within the article.
